# Supplementary material for: The human Dicer helicase domain is capable of ATP hydrolysis and single-stranded nucleic acid binding
Source: BMC Biol. 2024 Dec 18;22:287. doi: 10.1186/s12915-024-02082-x (PMC11658451; doi:10.1186/s12915-024-02082-x)
Supplement: Supplementary file 1 — Additional file 1: Fig. S1. Protein preparations used in the study. Fig. S2. ATP hydrolysis assay with the hDicer helicase domain (HEL) and the hDicer variant lacking the helicase domain (hDicer_ΔHEL). Fig. S3. Protein concentration-dependent ATP hydrolysis of hDicer_K70A and WT hDicer. Fig. S4. Protein concentration-dependent ATP binding of hDicer_K70A and WT hDicer. Fig. S5. Time-course of ATP hydrolysis by HEL and WT hDicer under high-turnover and low-turnover conditions. Fig. S6. ATP hydrolysis by HEL in the presence of competitors. Fig. S7. Nucleic acid binding activity of HEL in the absence and presence of nucleotides. Fig. S8. Nucleic acid binding activity of HEL measured using bio-layered interferometry (BLI). Fig. S9. Pre-miRNA binding by HEL or WT hDicer. Fig. S10. SASREF rigid body models of the hDicer helicase domain complexes with the pre-mir-16-1 and the pre-mir-21. Fig. S11. Double-stranded RNA (dsRNA) and double-stranded DNA (dsDNA) binding by HEL. Fig. S12. Nucleic acid binding activity of HEL in 4°C. Fig. S13. Denaturing PAGE analysis of 5′-32P-labeled R42 incubated with increasing amounts of HEL. Fig. S14. EMSA with HEL and 5ʹ-32P-labeled 42-nt (AC)21. Fig. S15. Secondary structures of ssRNAs and ssDNAs, used in the binding assays. Table S1. SAXS data collection and scattering-derived parameters. Table S2. Comparison of the theoretical and experimental thermodynamic parameters for R40 and the R40•HEL complex, calculated using Oligo Calc software (Biotools) (4) (theoretical) and circular dichroism (CD) spectroscopy (experimental). [file 12915_2024_2082_MOESM1_ESM.pdf]

The human Dicer helicase domain is capable of ATP hydrolysis and single-stranded nucleic acid binding

Kinga Ciechanowska[1], Agnieszka Szczepanska[1+], Kamil Szpotkowski[1+], Klaudia Wojcik[1], Anna Urbanowicz[2], Anna Kurzynska-Kokorniak[1*]

[1] Department of Ribonucleoprotein Biochemistry, Institute of Bioorganic Chemistry Polish Academy of Sciences, Zygmunta Noskowskiego 12/14, 61-704 Poznan, Poland

[2] Laboratory of Protein Engineering, Institute of Bioorganic Chemistry Polish Academy of Sciences, Zygmunta Noskowskiego 12/14, 61-704 Poznan, Poland

[+] These authors contributed equally to this work.

***** Correspondence: akurzyns@man.poznan.pl; Tel.: +48-61-852-8503 (ext. 1264)

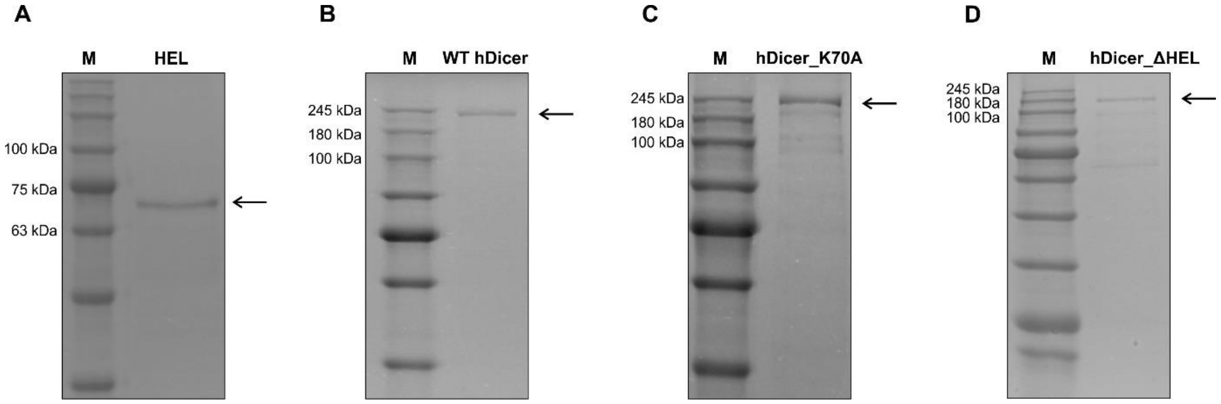

Fig. S1 Protein preparations used in the study. SDS-PAGE gel showing: **a** the hDicer helicase domain preparation (HEL), 1 µg; **b** the wild-type hDicer protein preparation (WT hDicer), 0.5 µg; **c** the hDicer variant with a mutation in the Walker A motif (hDicer_K70A), 1 µg; and **d** the hDicer variant lacking the helicase domain (hDicer_ΔHEL), 0.5 µg. The respective proteins are indicated with arrows. HEL was produced in *Escherichia coli* as a fusion to the His6-tag. WT hDicer, hDicer_K70A and hDicer_ΔHEL were produced in HEK 293T NoDice cells as fusions with the Flag-tag. M indicates protein mass marker (*Perfect*™ *Tricolor Protein Ladder*, EURx).

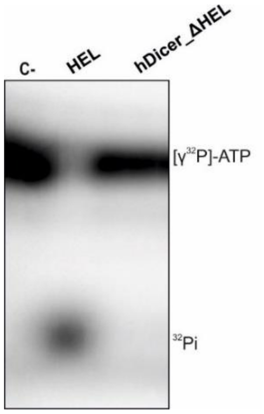

Fig. S2 ATP hydrolysis assay with the hDicer helicase domain (HEL) and the hDicer variant lacking the helicase domain (hDicer_ΔHEL). The reactions involved: HEL (2 nM) or hDicer_ΔHEL (2 nM) and [γ^{32}P]-ATP (2 nM), and were incubated at 37 °C for 30 min. C- indicates a control sample with no protein and ^{32}Pi indicates the product of [γ^{32}P]-ATP hydrolysis.

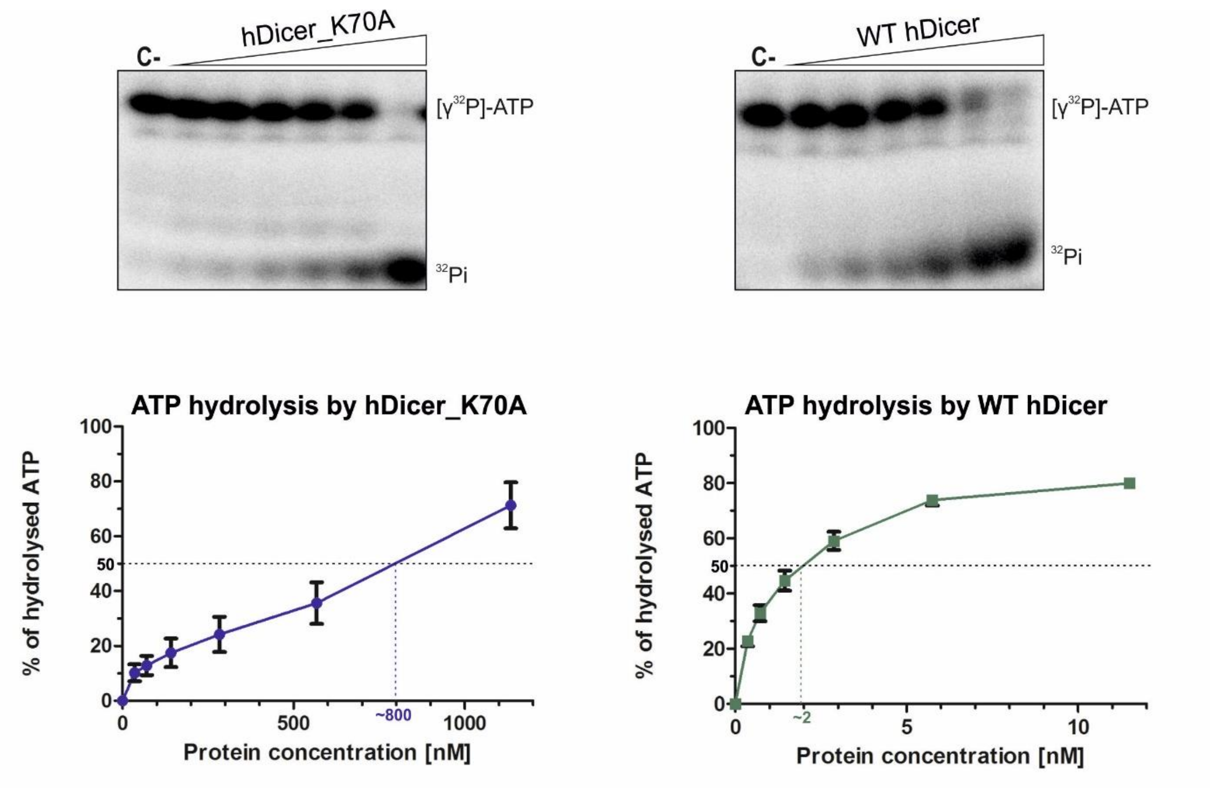

Fig. S3 Protein concentration-dependent ATP hydrolysis of hDicer_K70A and WT hDicer. PAGE analysis of the reaction mixtures containing: [γ^{32}P]-ATP (2 nM) and hDicer_K70A (36, 72, 144, 288, 575, 1150 nM) or WT hDicer (0.36, 0.72, 1.44, 2.88, 5.75, 11.5 nM), reaction mixtures were incubated 30 min at 37 °C. C- indicates a control sample with no protein and ^{32}Pi indicates the product of [γ^{32}P]-ATP hydrolysis. **Bottom panel** Quantitative analysis of the ATP hydrolysis assay. Error bars represent standard deviations (SD) based on three separate experiments.

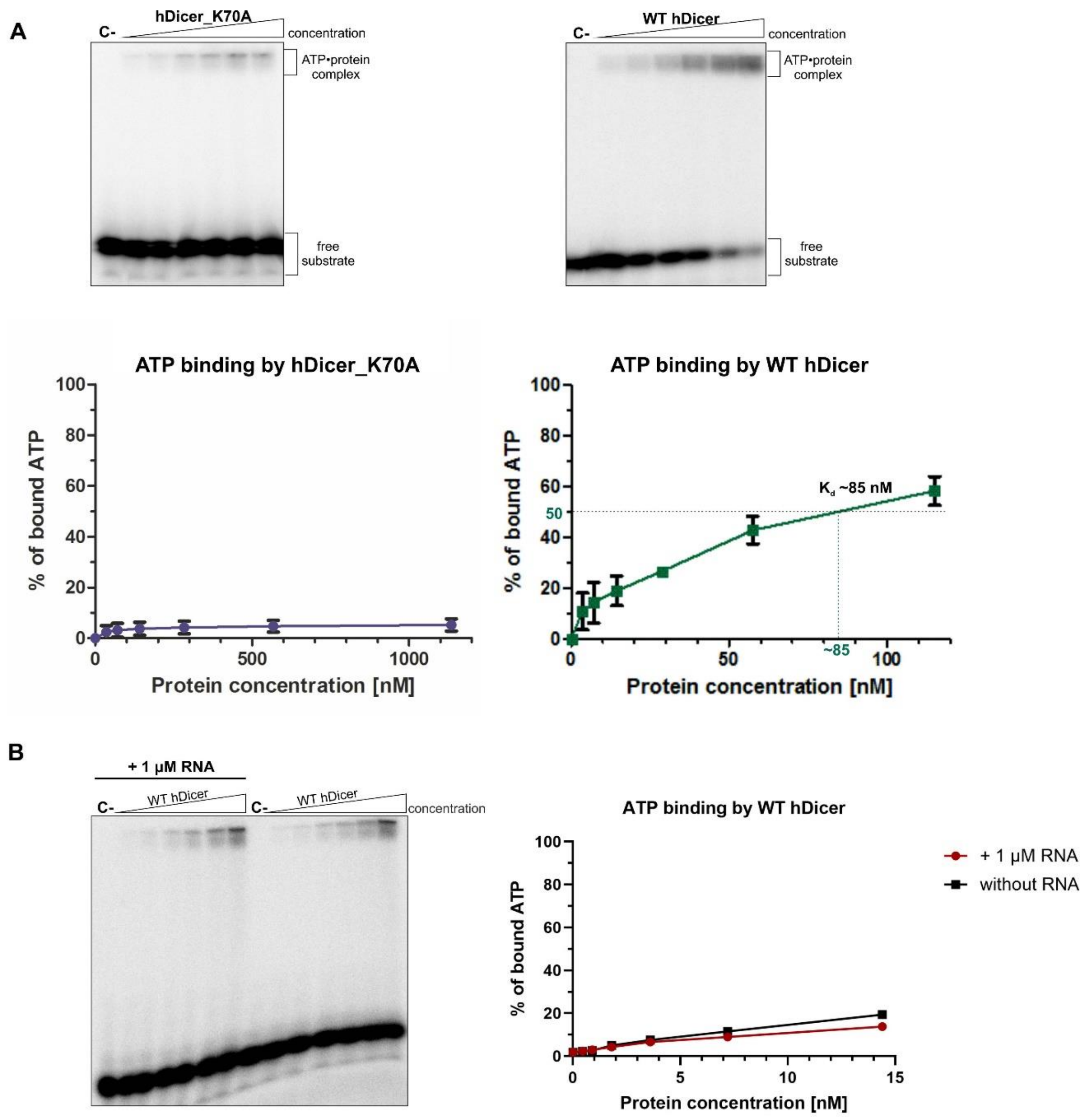

Fig. S4 Protein concentration-dependent ATP binding of hDicer_K70A and WT hDicer. **a** PAGE analysis of reaction mixtures containing: $[\gamma^{32}P]$-ATP (2 nM) and hDicer_K70A (36, 72, 144, 288, 575, 1150 nM) or WT hDicer (3.6, 7.2, 14.4, 28.8, 57.5, 115 nM), reaction mixtures were incubated 1 min at 4 °C. C- indicates a control sample with no protein. **Bottom panel** Quantitative analysis of the binding assay between hDicer_K70A or WT hDicer and ATP. Error bars represent SD from three separate experiments. **b** ATP binding by the wild-type hDicer (WT hDicer) with or without 1 μM non-labeled ssRNA. EMSA with $[\gamma^{32}P]$-ATP (2 nM) and increasing amounts of WT hDicer (0.45, 0.9, 1.8, 3.6, 7.2, 14.4 nM). Reaction mixtures were incubated at 4 °C for 1 min. C- indicates a control sample with no protein. **Right panel** Quantitative analysis of the binding assay between WT hDicer and ATP with or without 1 μM RNA.

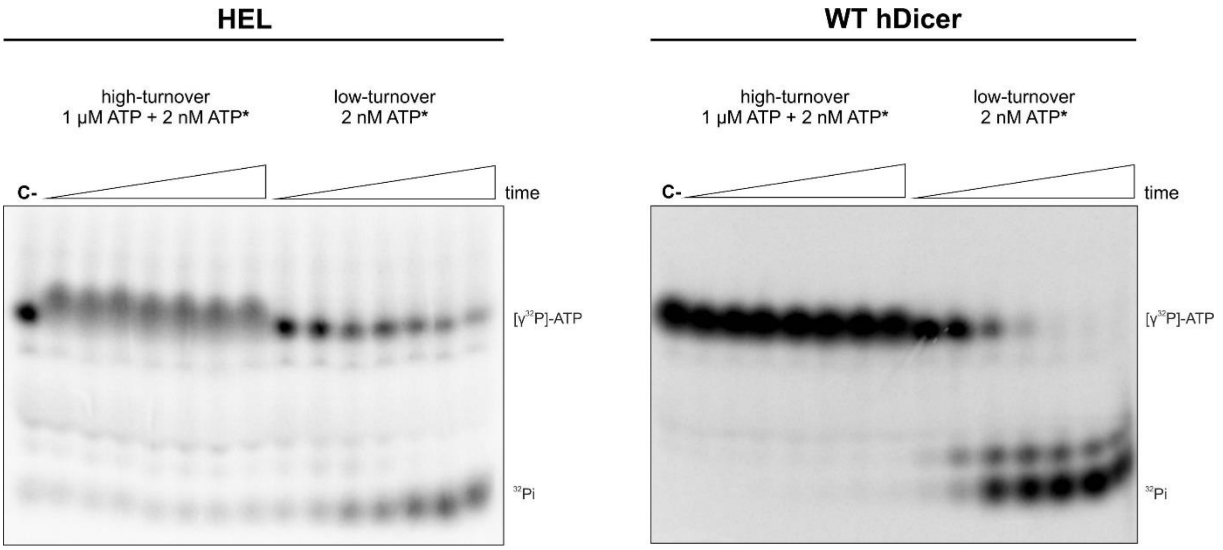

Fig. S5 Time-course of ATP hydrolysis by HEL and WT hDicer under high-turnover and low-turnover conditions. PAGE analysis of reaction mixtures containing (high-turnover): 1 µM ATP with [γ^{32}P]-ATP (2 nM) spiked in to monitor hydrolysis and HEL (2 nM) or WT hDicer (2 nM), (low-turnover): [γ^{32}P]-ATP (2 nM) and HEL (2 nM) or WT hDicer (2 nM). Reaction mixtures were incubated at 37 °C for: 1, 5, 15, 30, 60, 90 and 120 min. C- indicates a control sample with no protein and ^{32}Pi indicates the product of [γ^{32}P]-ATP hydrolysis.

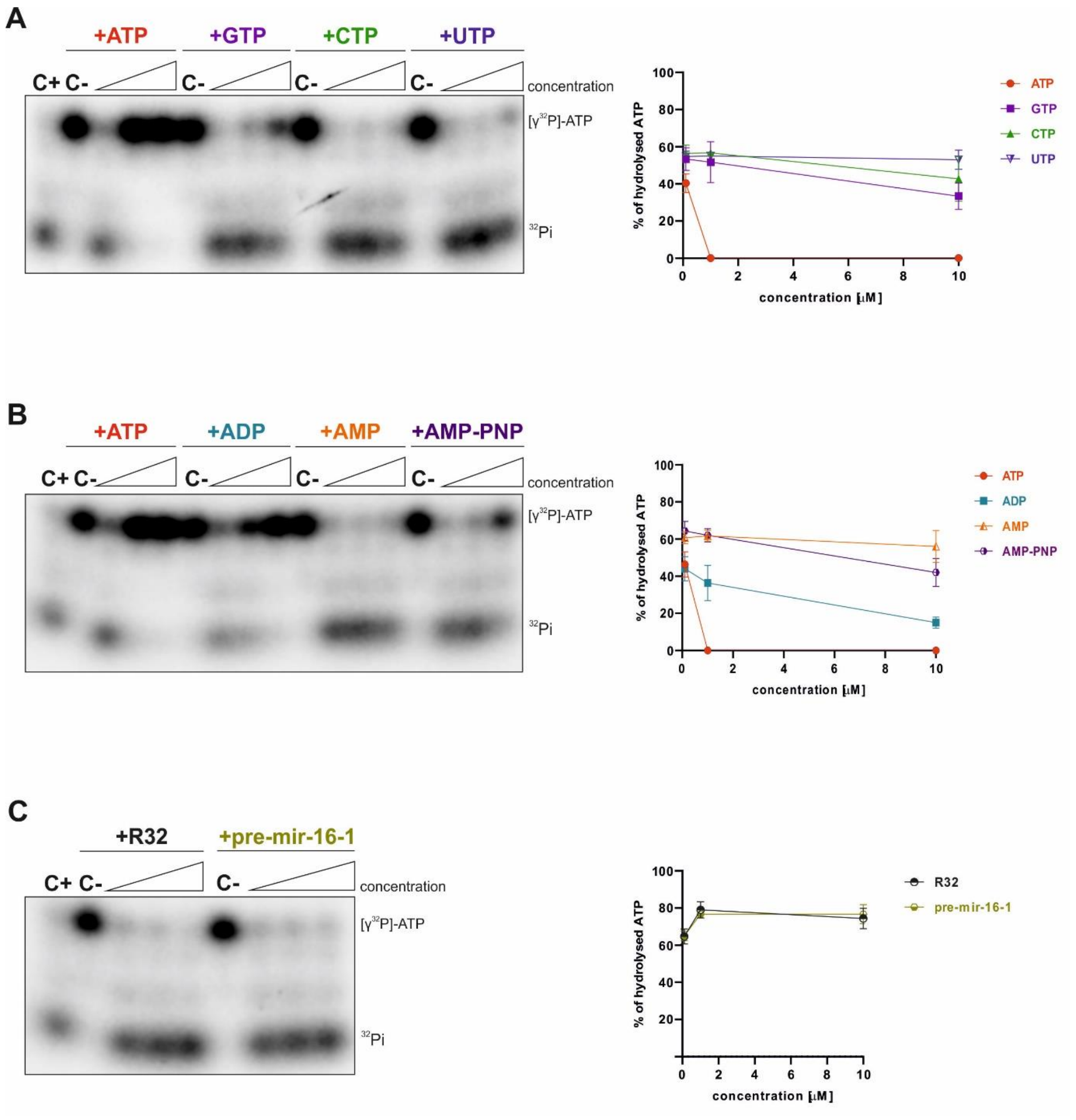

Fig. S6 ATP hydrolysis by HEL in the presence of competitors. PAGE analysis of reaction mixtures containing: **a** [γ^{32}P]-ATP (2 nM), HEL (2 nM) and non-labeled competitors: ATP, GTP, CTP or UTP (0.01, 1, 10 µM), **b** [γ^{32}P]-ATP (2 nM), HEL (2 nM) and non-labeled competitors: ATP, ADP, AMP or a non-hydrolyzable analogue of ATP, AMP-PNP (0.01, 1, 10 µM), **c** [γ^{32}P]-ATP (2 nM), HEL (2 nM) and non-labeled competitors: 32-nt RNA (R32) or pre-mir-16-1 (0.01, 1, 10 µM). Reaction mixtures were incubated at 37 °C for 30 min. C- indicates a control sample with no protein. C+ indicates a positive control: [γ^{32}P]-ATP (2 nM) and HEL (2 nM) incubated at 37 °C for 30 min, for C+ the ATP hydrolysis was ~ 60%. ^{32}Pi indicates the product of [γ^{32}P]-ATP hydrolysis. **Right panels** Quantitative analysis of the ATP hydrolysis assays. Error bars represent standard deviations (SD) based on three separate experiments.

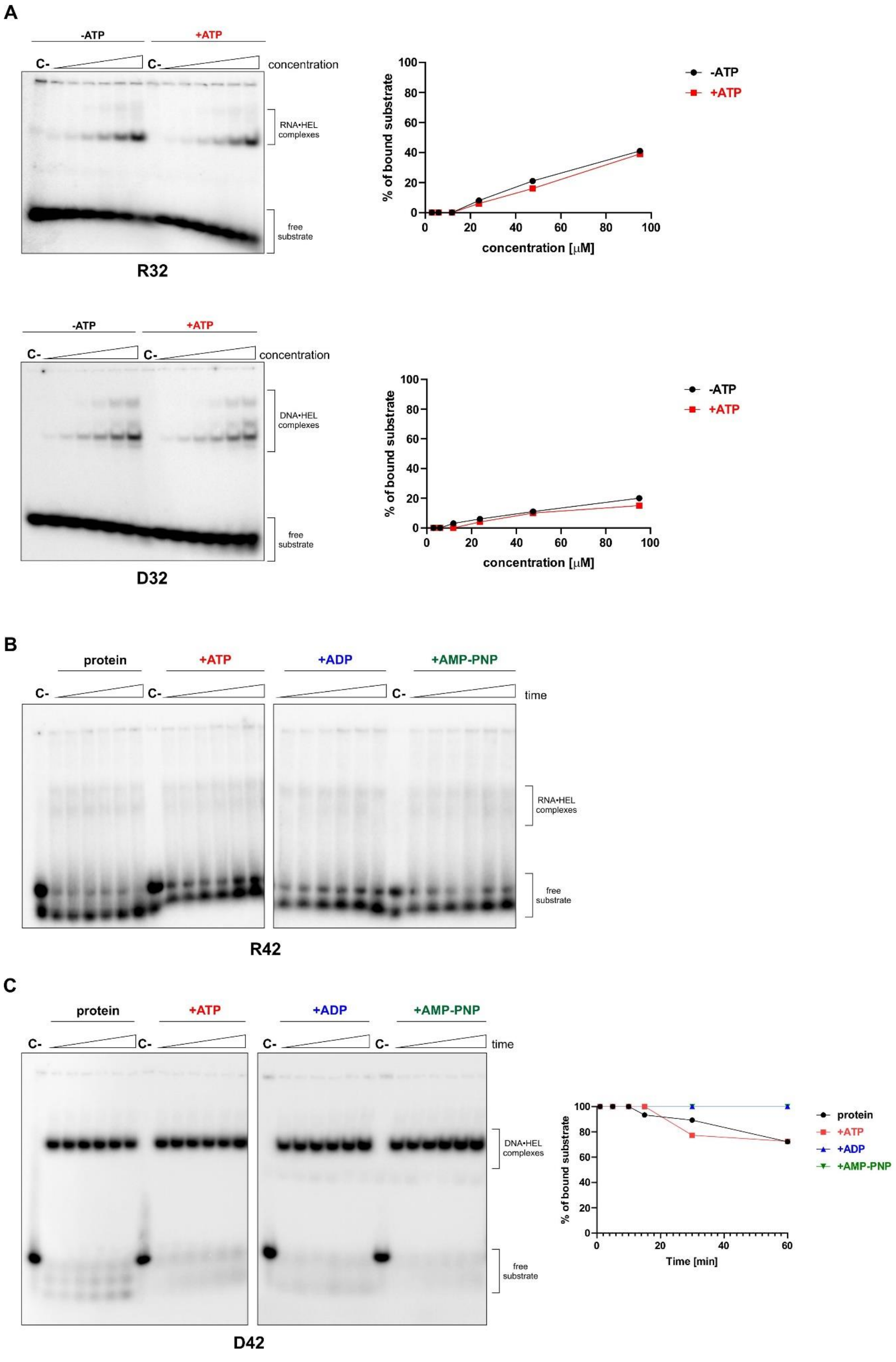
A
-ATP
+ATP
C-
C-
concentration
RNA•HEL complexes
free substrate
R32
% of bound substrate
concentration [μM]
-ATP
+ATP
-ATP
+ATP
C-
C-
concentration
DNA•HEL complexes
free substrate
D32
% of bound substrate
concentration [μM]
-ATP
+ATP
B
protein
+ATP
+ADP
+AMP-PNP
C-
C-
C-
time
RNA•HEL complexes
free substrate
R42
C
protein
+ATP
+ADP
+AMP-PNP
C-
C-
C-
C-
time
DNA•HEL complexes
free substrate
D42
% of bound substrate
Time [min]
protein
+ATP
+ADP
+AMP-PNP

Fig. S7 Nucleic acid binding activity of HEL in the absence and presence of nucleotides. **a** EMSA with HEL, 5′-^{32}P-labeled R32 or D32 (2.5 nM) and ± ATP (1 mM). Increasing amounts of HEL (2.97, 5.94, 11.86, 23.75, 47.5, 95 μM) are represented by a triangle. Reaction mixtures were incubated at room temperature for 15 min. C- indicates a control sample with no protein. **Right panels** Quantitative analysis of the binding assay between HEL and R32 or D32. The x-axis represents the HEL concentration, and the y-axis represents the percentage of bound substrate by HEL. **b** EMSA with HEL (25 μM) and 5′-^{32}P-labeled 42-nt RNA (R42) (2.5 nM). **c** EMSA with HEL (25 μM) and 5′-^{32}P-labeled 42-nt DNA (D42) (2.5 nM). The binding reactions were carried out in the absence of nucleotide or the presence of ATP, ADP or AMP-PNP (1 mM). Samples were analyzed at: 1, 5, 10, 15, 30 and 60 min. Reaction mixtures were incubated at room temperature. C- indicates a control sample with no protein. **Right panel** Quantitative analysis of the time-course binding assay between HEL and D42. The x-axis represents the incubation time expressed in minutes, and the y-axis represents the percentage of bound substrate by HEL.

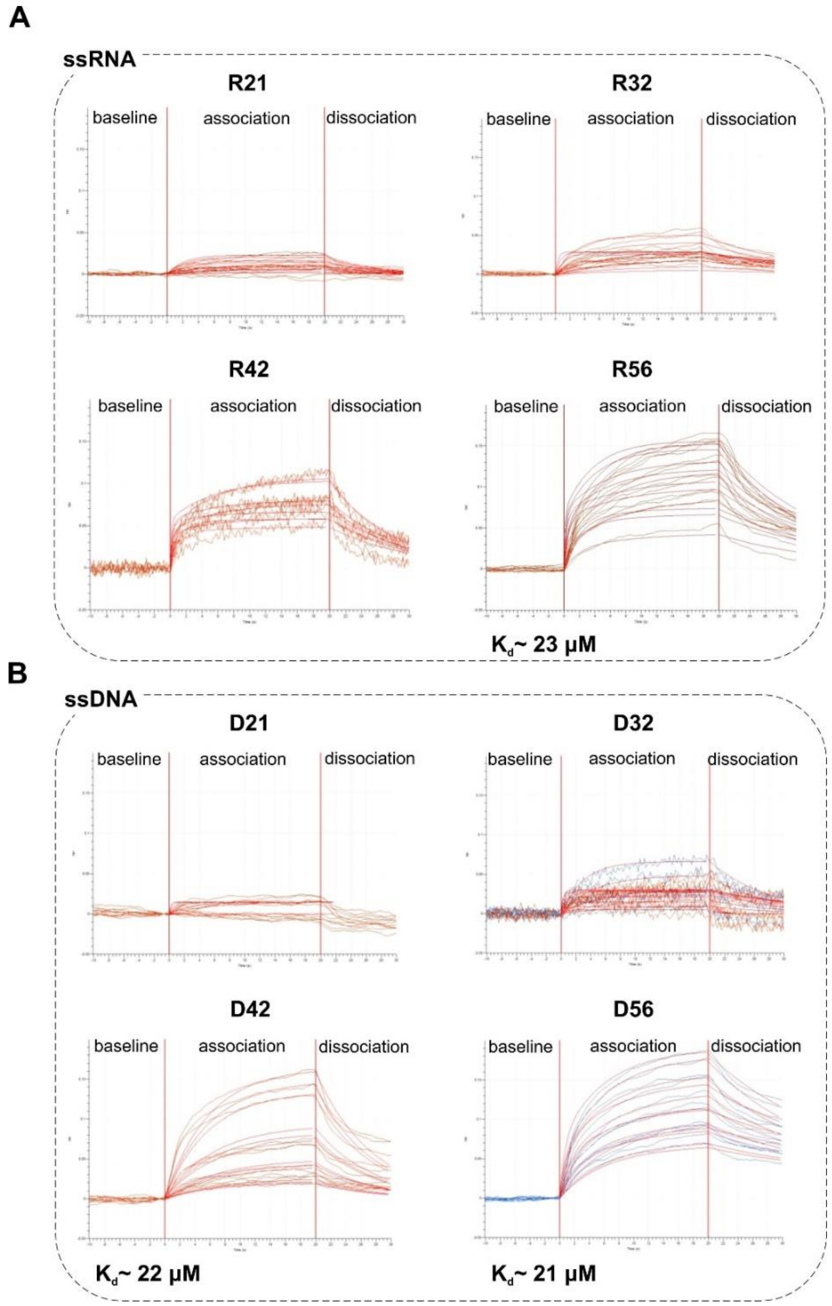

Fig. S8 Nucleic acid binding activity of HEL measured using bio-layered interferometry (BLI). Binding curves obtained by BLI for **a** ssRNAs and **b** ssDNAs and HEL. In the experiment, HEL (1 μM) was incubated with increasing amounts of ssRNA (3.125, 6.25, 12.5, 25, 50, 100 μM): R21, R32, R42, R56; or increasing amounts of ssDNA (3.125, 6.25, 12.5, 25, 50 100 μM): D21, D32, D42, D56. Measurements were carried out at 23 °C.

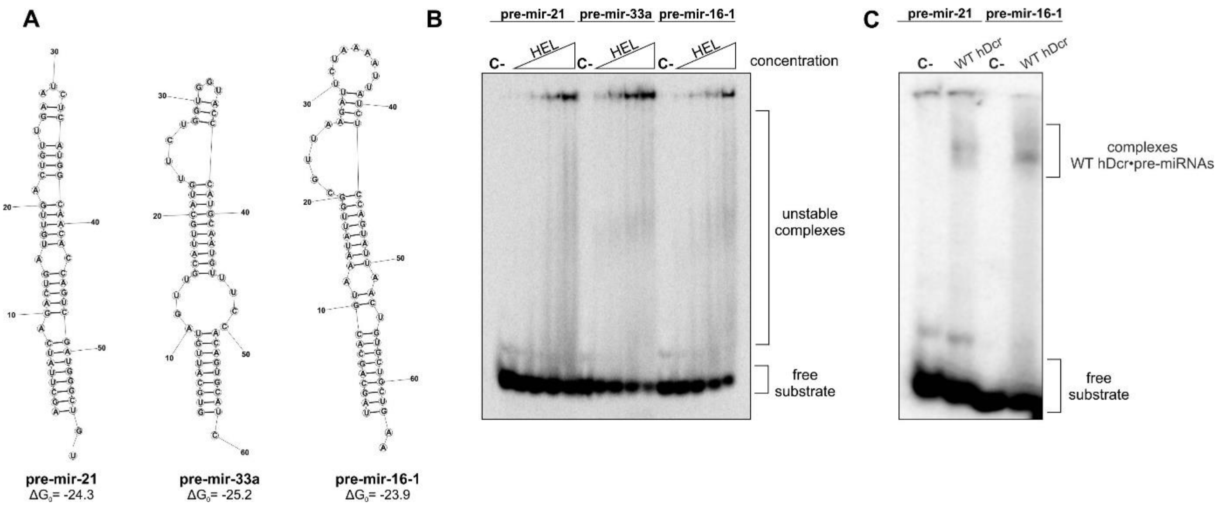

Fig. S9 Pre-miRNA binding by HEL or WT hDicer. **a** Secondary structures of pre-miRNAs used in the study. The predicted structures for pre-mir-21, pre-mir-33a, and pre-mir-16-1 generated using the RNAstructure Fold online tool (Mathews Lab). Free energy values expressed in kcal/mol are shown at the bottom. Nucleotides are numbered starting from the 5'-end. **b** EMSA with HEL and 5′-^{32}P-labeled pre-miRNAs (2.5 nM): pre-mir-21, pre-mir-33a, pre-mir-16-1. Increasing amounts of HEL (11.86, 23.75, 47.5, 95 µM) are represented by a triangle. Reaction mixtures were incubated at room temperature for 15 min. C- indicates a control sample with no protein. **c** EMSA with WT hDicer, hDcr (10 µM) and 5′-^{32}P-labeled pre-miRNAs (2.5 nM) pre-mir-21 or pre-mir-16-1. Reaction mixtures were incubated at room temperature for 15 min. C- indicates a control sample with no protein.

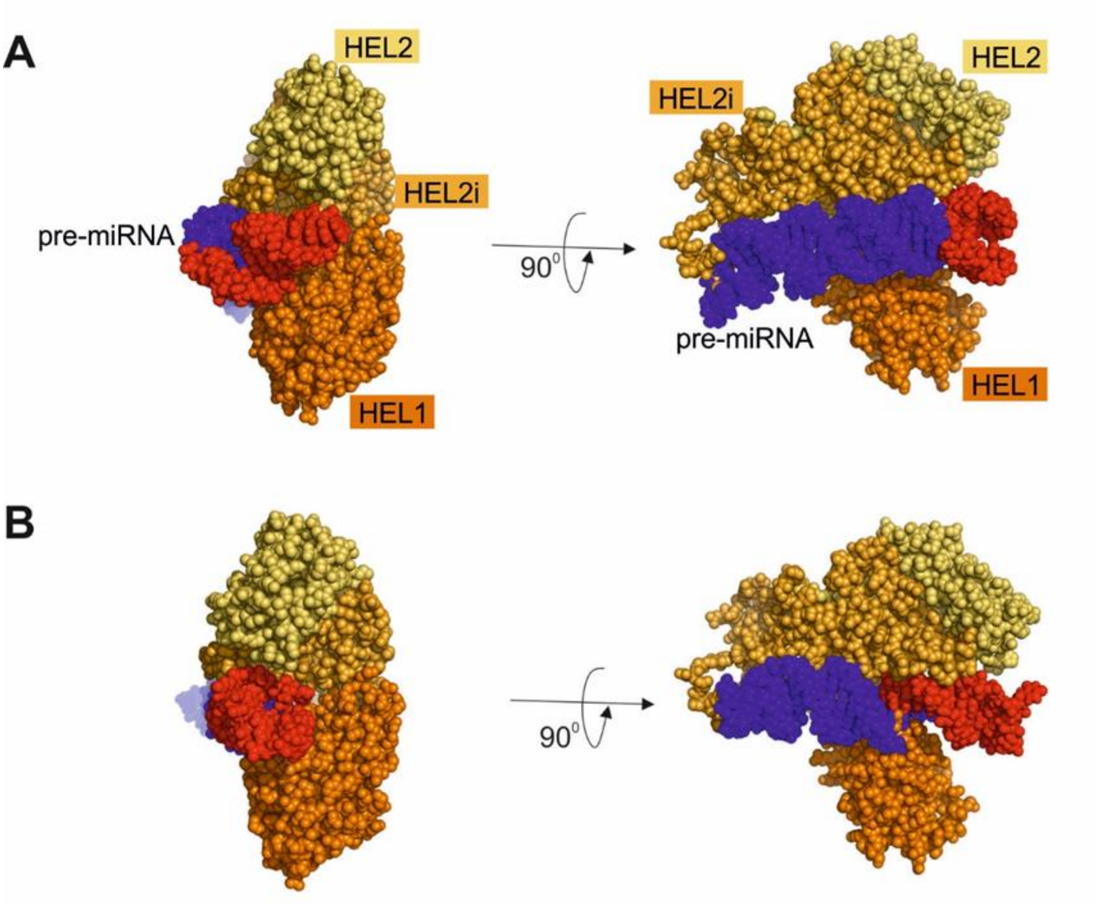

Fig. S10 SASREF rigid body models of the hDicer helicase domain complexes with **a** pre-mir-16-1 and **b** pre-mir-21. Structure of the hDicer helicase domain (PDB entry 5ZAL) was generated using SWISS MODEL and structures of pre-miRNAs were predicted using RNAComposer. The three helicase subdomains are distinguished: HEL1 (bright orange), HEL2i (light orange), HEL2 (yellow). In the pre-miRNA structures, stem regions are indicated in purple, while the apical loop regions (nucleic acid residues 20-40) are indicated in red.

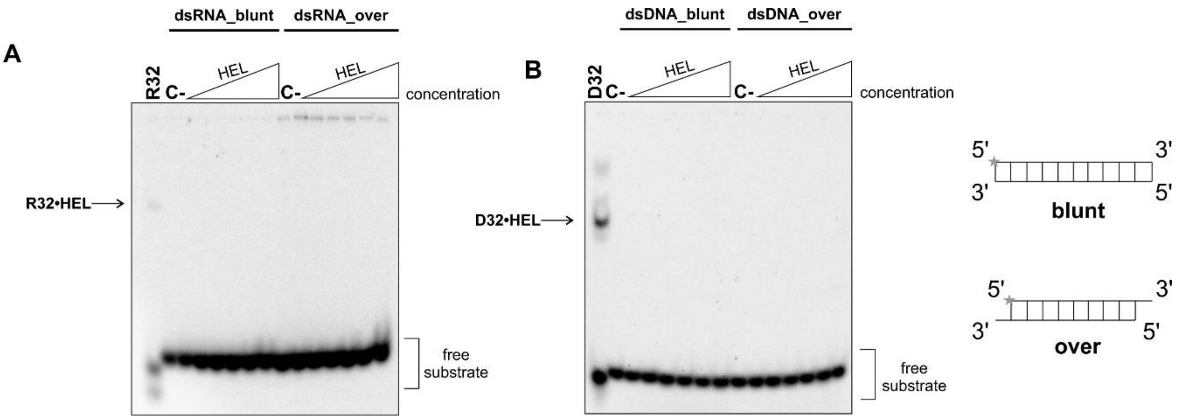

Fig. S11 Double-stranded RNA (dsRNA) and double-stranded DNA (dsDNA) binding by HEL. **a** EMSA with HEL and ^{32}P-labeled 32-bp RNA (dsRNA_blunt) (2.5 nM) and 30-bp RNA duplex with a 2-nt 3′ overhang on each end (dsRNA_over) (2.5 nM), R32 – a control sample: ^{32}P-labeled R32 incubated with 95 µM HEL. **b** EMSA with HEL and ^{32}P-labeled 32-bp DNA (dsDNA_blunt) (2.5 nM) and 30-bp DNA duplex with a 2-nt 3′ overhang on each end (dsRNA_over) (2.5 nM), D32 – a control sample: ^{32}P-labeled D32 incubated with 95 µM HEL. Increasing amounts of HEL (2.97, 5.94, 11.86, 23.75, 47.5, 95 µM) are represented by a triangle. Reaction mixtures were incubated at room temperature for 15 min. C- indicates a control sample with no protein.

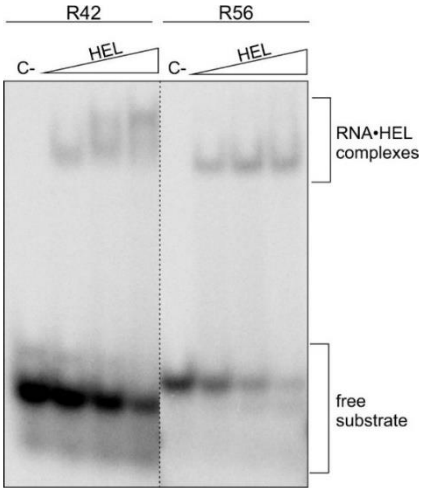

Fig. S12 Nucleic acid binding activity of HEL in 4°C. EMSA with HEL and 5′-^{32}P-labeled single-stranded RNAs (ssRNAs) (2.5 nM): R42, R56. Increasing amounts of HEL (23.75, 47.5, 95 µM) are represented by a triangle. Reaction mixtures were incubated at 4°C for 15 min. C- indicates a control sample with no protein.

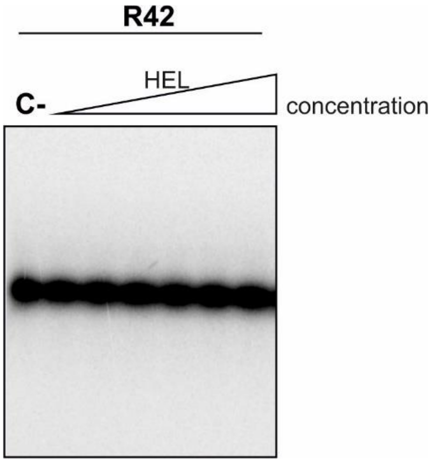

Fig. S13 Denaturing PAGE analysis of 5′-^{32}P-labeled R42 (2.5 nM) incubated with increasing amounts of HEL (2.97, 5.94, 11.86, 23.75, 47.5, 95 μM). Reaction mixtures were incubated for 15 min at 37 °C, then they were denatured at 95 °C in a loading buffer containing 10 M urea, and separated in an 8% denaturing (7 M urea) PAA gel. C- indicates a control sample with no protein.

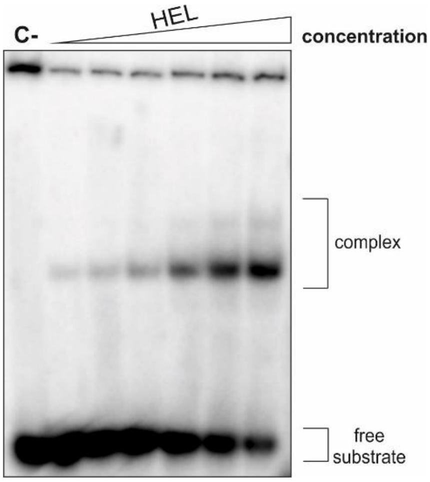

Fig. S14 EMSA with HEL and 5′-^{32}P-labeled 42-nt $(AC)_{21}$ (2.5 nM). Increasing amounts of HEL (2.97, 5.94, 11.86, 23.75, 47.5, 95 μM) are represented by a triangle. Reaction mixtures were incubated at room temperature for 15 min. (C-) − a control sample with no protein.

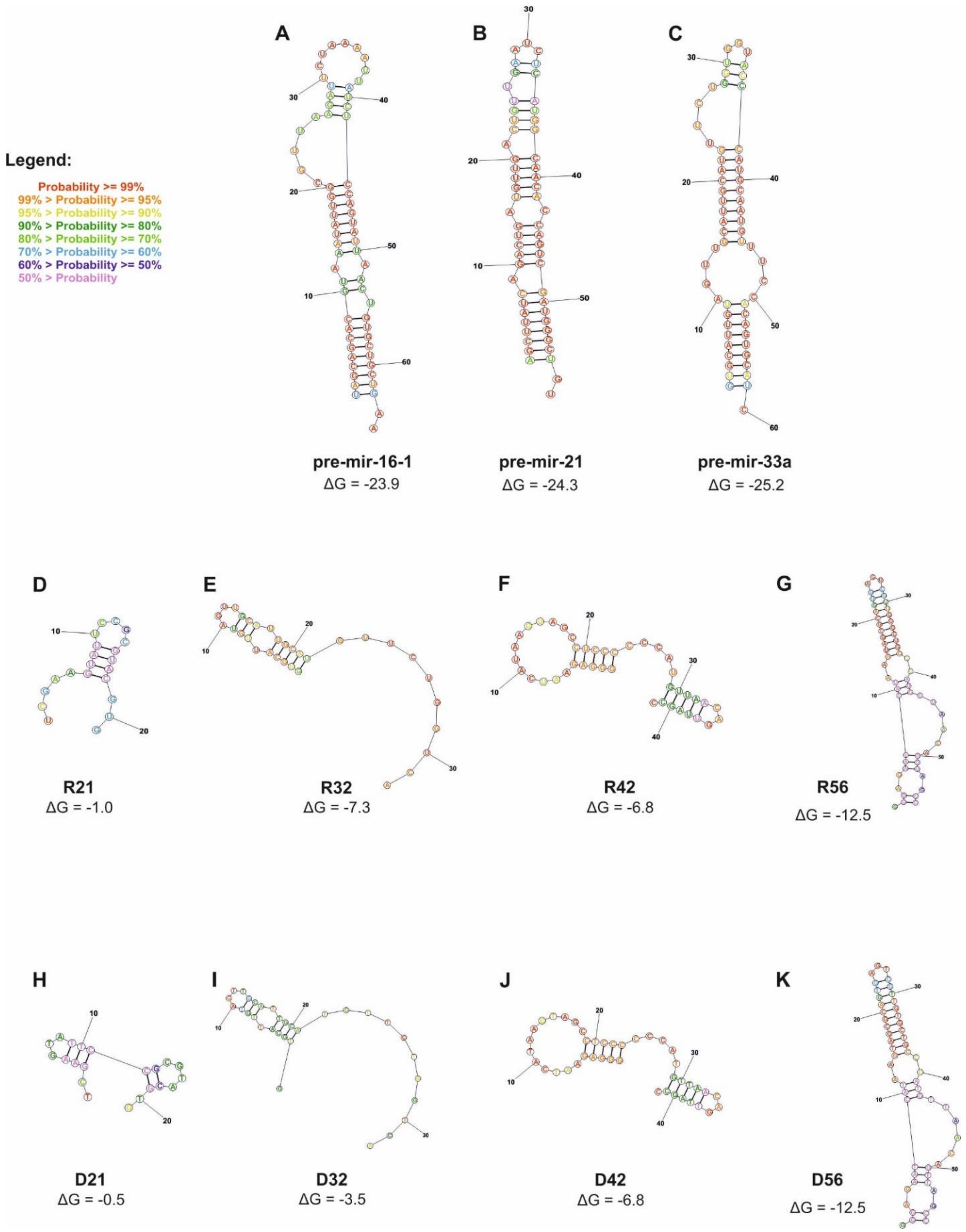

Fig. S15 Secondary structures of ssRNAs and ssDNAs, used in the binding assays (**Fig. 5**), generated using the RNAstructure Fold online tool (Mathews Lab). The free energy value expressed in kcal/mol is shown at the bottom. Nucleotides are numbered starting from the 5'-end.

Table S1 SAXS data collection and scattering-derived parameters.

	HEL	pre-mir-21•HEL	pre-mir-16-1•HEL	pre-mir-21	pre-mir-16-1
Data collection					
Instrument	P12 beamline PETRA III storage ring				
Wavelength (Å)	1.24				
S range (nm^{-1})	0.0088-5				
Exposure time (s)	1				
Temperature (K)	293				
Structural parameters					
I_0 (arbitrary units) [from P(r)]	360.93	410.72	406.7	-	-
R_g (Å) [from p(r)]	34.26	37.93	38.18	-	-
I_0 (arbitrary units) (from Guinier region)	360.93	410.75	405.6	-	-
R_g from crystal structure	32.45	-	-	28.30	28.77
Porod volume estimate (Å^3)	84394	111284	115287	-	-
Dry volume calculated from sequence (Å^3)	92970	109840	111840	16870	18870
Molecular-mass determination					
Contrast ($\Delta\rho x10^{10}$ cm^{-2})	3.047	3.047	3.047	3.047	3.047
Molecular mass M_w [from I(0)] (kDa)	74689	94268	95605	-	-
Molecular mass from sequence	71462	89156	91202	17664	19740
Software used					
Primary data reduction	PRIMUS				
Data processing	PRIMUS				
Quaternary structure modeling	SASREF				
Computation of model intensities	CRYSOL				
Three dimensional graphics representation	PyMOL				

Table S2 Comparison of the theoretical and experimental thermodynamic parameters for R40 and the R40•HEL complex, calculated using Oligo Calc software (Biotools) (theoretical) and circular dichroism (CD) spectroscopy (experimental).

	Molecular mass of RNA [Da]	GC content [%]	T_{mbasic} [°C]	T_{msalt} [°C]	$T_{m\ exp.}$ [°C]	RlnK [cal/(°Kmol)]	ΔG [kcal/mol]	$\Delta G_{exp.}$ [kcal/mol]	ΔH [kcal/mol]	$\Delta H_{exp.}$ [kcal/mol]	ΔS [cal/(°Kmol)]	$\Delta S_{exp.}$ [cal/(°Kmol)]
R40	12741	50	69,4	79,9	35/75	33,404	58,6	56,7	347,5	336,03	915,5	885,28
R40•HEL hDicer	12741	50	69,4	79,9	60	33,404	58,6	50,2	347,5	297,81	915,5	784,58

T_{mbasic} – basic melting temperature

T_{msalt} – salt-adjusted melting temperature

$T_{m\ exp.}$ – experimental melting temperature

RlnK – ratio: R – gas constant (ok. 8,31 [J*mol^{-1}*K^{-1}]) and natural logarithm from K – equilibrium constant [mol/L]

ΔG – Gibbs energy

$\Delta G_{exp.}$ – experimental Gibbs energy

ΔH – theoretical enthalpy

$\Delta H_{exp.}$ – experimental enthalpy

ΔS – theoretical entropy

$\Delta S_{exp.}$ – experimental entropy
