# Supplementary material for: The human Dicer helicase domain is capable of ATP hydrolysis and single-stranded nucleic acid binding
Source: BMC Biol. 2024 Dec 18;22:287. doi: 10.1186/s12915-024-02082-x (PMC11658451; doi:10.1186/s12915-024-02082-x)
Supplement: Supplementary file 2 — Additional file 2. Datasets related to the SAXS studies. [file 12915_2024_2082_MOESM2_ESM.pdf]

Datasets related to the SAXS studies

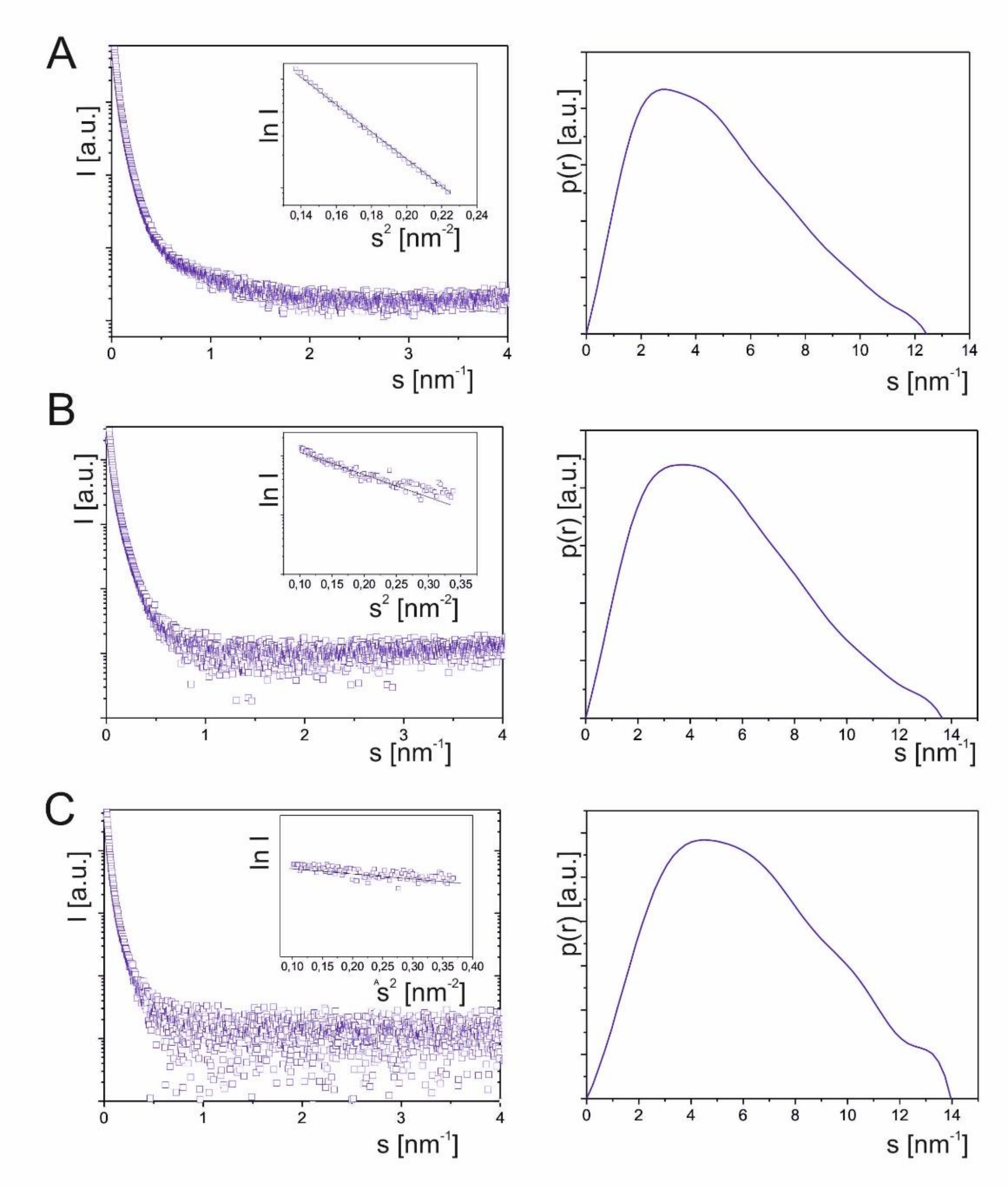

Experimental SAXS data. Plots present data for: **a** HEL, **b** HEL•pre-mir-21 complex, and **c** HEL•pre-mir-16-1 complex. Experimental SAXS curves (left) and Guinier plots (insert). A straight line in a Guinier region suggests that the system is essentially monodisperse. Pair distance distribution function, the *p(r)* function (right). The *p(r)* function is a histogram of all pairwise distances *r* between two scattering elements in the macromolecules weighted by their electron density contrast. The *p(r)* function is a representation of the shape of the molecule (or the complex of molecules) in real space. In the case of HEL and HEL complexes with pre-miRNAs, the *p(r)* function shows an elongated shape of the analyzed molecules.
